# Patient Survival and Its Relation to Tumor Absorbed Dose in [^177^Lu]Lu-DOTATATE Therapy of Neuroendocrine Tumors

**DOI:** 10.2967/jnumed.125.271439

**Published:** 2026-10

**Authors:** Carl Fredrik Warfvinge, Selma Curkic Kapidzic, Johan Gustafsson, Lovisa Jessen, Johanna Svensson, Anna Sundlöv, Katarina Sjögreen Gleisner

**Affiliations:** 1 Department of Haematology, Oncology, and Radiation Physics, Skåne University Hospital, Lund, Sweden;; 2 Division of Oncology, Department of Clinical Sciences Lund, Lund University, Lund, Sweden;; 3 Medical Radiation Physics, Lund, Lund University, Lund, Sweden; and; 4 Department of Oncology, Institute of Clinical Sciences, Sahlgrenska Academy, University of Gothenburg, Gothenburg, Sweden

**Keywords:** oncology, endocrine, dosimetry, neuroendocrine tumor

## Abstract

[^177^Lu]Lu-DOTATATE is an established treatment for metastasized neuroendocrine tumors (NETs). It is postulated that a higher tumor absorbed dose (AD) improves clinical efficacy. The aim of this study was to assess the average AD of the entire tumor burden and analyze its effect on survival outcomes. **Methods:** This post hoc analysis included 81 patients with metastasized grade 1 and grade 2 NETs treated with [^177^Lu]Lu-DOTATATE individualized based on renal dosimetry. Tumor ADs were quantified using quantitative SPECT imaging and mass-weighted averaging across all lesions. Patients were stratified into high- and low-AD groups based on median ADs for cycle 1 and cumulative AD over all cycles. Survival outcomes were assessed using Kaplan–Meier estimates and Cox proportional hazards models. **Results:** Higher tumor AD was significantly associated with improved progression-free survival and overall survival. In multivariable analysis, patients in the high-AD group had significantly lower hazard ratios (HRs) for progression-free survival (HR, 0.34 for cycle 1; HR, 0.37 for cumulative AD) and overall survival (HR, 0.41 for cycle 1; HR, 0.48 for cumulative AD). Patients with a cumulative AD of 118 Gy or more (top quartile) had significantly improved survival compared with the remaining cohort, without increased toxicity. **Conclusion:** Mean tumor AD to the entire tumor burden is an independent predictor of survival in patients with grade 1 and grade 2 NETs treated with [^177^Lu]Lu-DOTATATE. These findings support the use of tumor dosimetry in treatment planning and suggest that a target AD of around 118 Gy is clinically relevant.

## Visual Abstract

**Figure:**
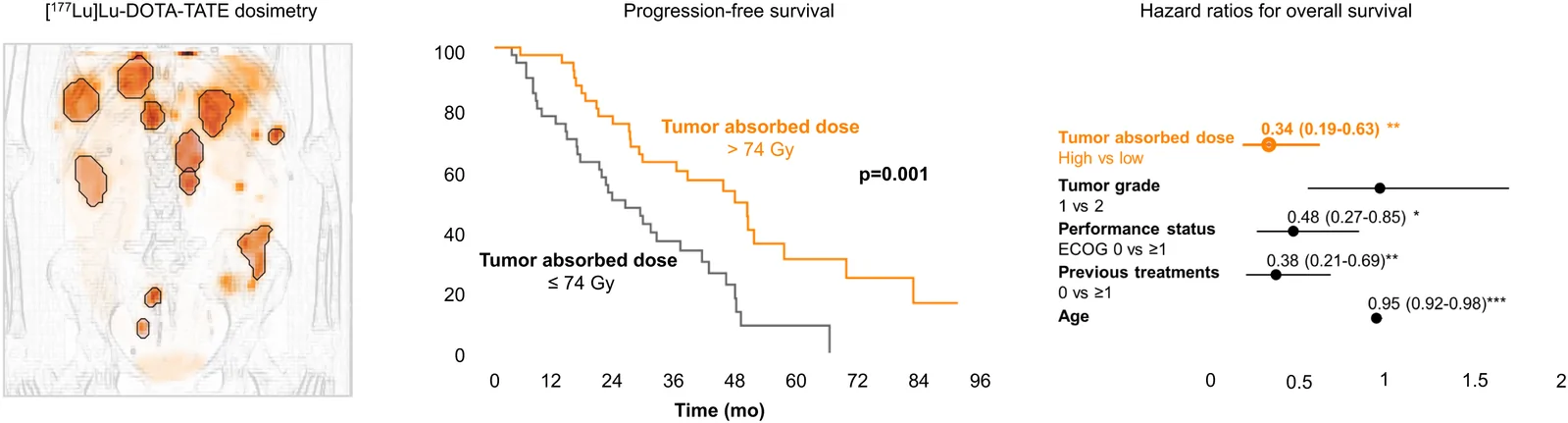

Peptide receptor radionuclide therapy (PRRT) with [^177^Lu]Lu-DOTATATE is an established treatment for patients with advanced neuroendocrine tumors (NETs), particularly gastroenteropancreatic NETs. Its approval is based on the NETTER-1 trial, in which all patients received 4 cycles of 7.4 GBq (1). It has long been proposed that individualizing the administered activity through dosimetry could enhance patient outcomes. A few studies have explored this treatment approach by tailoring the cumulative administered activity based on the absorbed dose (AD) to organs at risk. Although this strategy has proven feasible, it has not yielded significant clinical benefits compared with the standardized protocol used in NETTER-1 (2–4).

More recently, attention has shifted toward the role of the AD to tumors in predicting clinical outcomes, with several independent studies reporting correlations between tumor AD and clinical outcomes, including both tumor response (5–9) and patient survival (10–12). When considered together, these studies are informative. However, they share limitations such as small sample size, lack of multivariable survival analysis, short follow-up duration, and dosimetric assessment based on selected lesions rather than the entire tumor burden, thereby potentially introducing selection bias and reducing reproducibility. Hence, it is necessary to further investigate the relationship between tumor AD and survival with focus on long-term follow-up and adjustment for confounding factors.

The aims of the present study were to explore the relationship between the average tumor AD of the whole tumor burden and survival outcomes and to identify a target AD for subsequent studies, through post hoc analysis of data from a prospective clinical trial.

## MATERIALS AND METHODS

### Patient Data

Data were obtained from the ILUMINET trial (NCT01456078) (2), a phase 2, nonrandomized clinical study conducted at 2 sites in Sweden between 2011 and 2019. The trial was approved by the institutional review board, and all patients provided written informed consent. In total, 103 patients with somatostatin receptor–positive NETs were enrolled. Eligible patients were required to have a Ki-67 index of no more than 20%, as determined from the most recent biopsy before study entry. Patients received repeated cycles of [^177^Lu]Lu-DOTATATE (7.4 GBq) administered every 8–12 wk. Kidney dosimetry was performed for each cycle based on the combination of planar scintigraphy at 4 time points until 7 d after administration and 1 SPECT/CT at day 1. Treatment stopped at a cumulative renal biologically effective dose of 27 Gy. However, patients less than 70 y old with preserved renal and hematologic function and no progression could continue to 40 Gy. Trial follow-up continued until November 30, 2019. Progression-free survival (PFS) was measured from study inclusion to radiologic progression (RECIST 1.1) (13) or death and overall survival (OS) from inclusion to death. The overall results of the trial have been previously published (4).

For the present analysis, all patients who had completed treatment according to the protocol were deemed eligible for inclusion.

### Tumor Dosimetry

The mean AD across all tumors was quantified for each cycle, where the main support for tumor identification was the [^177^Lu]Lu-DOTATATE uptake exhibited in peritherapeutic SPECT images. γ-cameras were equipped with medium-energy collimators, and projections were acquired for an energy window of around 208 keV. Quantitative SPECT reconstruction was made using the methods embedded in the LundADose software suite (14,15), including compensations for attenuation, scatter, and collimator response (16).

Tumors were identified and delineated in the SPECT images using an automated method. The method relied on difference of gaussian filtering, which is an established image-processing technique for blob detection; see Supplement 1 in the supplemental materials (available at http://jnm.snmjournals.org) (17). For the first cycle, all generated tumor volumes of interest (VOIs) were inspected by the responsible oncologist. This visual inspection was made on the basis of the contrast-enhanced CT or MR images acquired closest in time to the respective SPECT image, confirming that the resulting VOIs represented tumor tissue. When necessary, VOIs were manually added, removed, or adjusted. For remaining cycles, tumor selection was restricted to the set of approved VOIs from cycle 1 by using them as reference for coregistration of the tumor VOIs defined for other cycles and discarding any VOIs that did not correspond to those of cycle 1. The result of this segmentation was visually evaluated by an experienced medical physicist with support from the responsible oncologist. Partial-volume correction was performed on an individual tumor basis, using recovery coefficients with the VOI volume for each tumor as input (18). The activity concentration was converted to AD rate by the assumption of local energy deposition of the emitted electron energy from ^177^Lu (19). ADs were calculated using the global effective half-lives previously obtained for a subset of the included patients, using an effective half-life of 103 h for grade 1 NETs, and 81 h for grade 2 NETs (18). The mean AD across all tumors for a given cycle was determined as the mass-weighted average, by taking the product of the individual tumor AD and its volume assuming a density of 1 g/mL, summing over all tumors, and dividing by the total tumor mass. Essentially this was equivalent to forming the quotient of the total radiation energy deposited in tumors and the total tumor mass (20). The cumulative tumor AD over all cycles was calculated by summation of the mean ADs for individual cycles.

### Relationship Between AD and Survival

Patients were categorized into low-AD or high-AD groups, based on the mean tumor ADs delivered during cycle 1 and cumulatively across all cycles, respectively. For both analyses, the medians across all patients were used for categorization (higher vs. lower than the median AD), yielding AD groups with equal number of patients. Kaplan–Meier estimates of PFS and OS and log-rank tests were applied to evaluate differences in survival between groups. Hazard ratios (HRs) and 95% CI for death and disease progression were calculated with Cox regression proportional hazards modeling in unadjusted analysis and in a multivariable model.

Variables included in the multivariable Cox regression model were selected on the basis of a directed acyclic graph constructed to visualize potential causal relationships (Supplement 2). Factors deemed to have the highest clinical relevance were chosen. The number of covariates was limited to approximately 1 variable per 10 events. To reduce the number of comparisons and improve model stability, performance status and the number of prior treatments were dichotomized (0 vs. ≥1). Tumor origin and tumor grade were found to be correlated (Supplement 3). Therefore, only tumor grade was included in the primary model. A sensitivity analysis in which tumor grade was replaced by origin, dichotomized as siNET versus non-siNET, was performed, yielding comparable results (Supplement 3). To enable direct comparison between PFS and OS, the same 5 clinical variables were included in both multivariable models. The final model consisted of tumor grade (1 vs. 2), performance status (Eastern Cooperative Oncology Group [ECOG] performance status 0 vs. ≥1), age (continuous), previous treatment lines other than long-acting somatostatin analogs (SSAs) (0 vs. ≥1), and tumor AD (higher vs. lower than the median AD). The proportional hazards assumption was verified for all included variables by inspecting log–log survival curves. Categoric variables were defined explicitly in SPSS (IBM) using indicator coding, with the first category set as the reference. The model was constructed using the Enter method. A *P* value of 0.05 or lower was considered significant. All statistical analyses were performed using SPSS version 30.0.0.0.

### Identification of a Clinically Relevant Threshold for AD

To identify an AD that could serve as a clinically useful target in future treatment planning, the cohort was further separated into quartiles on the basis of the AD delivered in cycle 1 and cumulatively across all cycles, respectively. Kaplan–Meier estimates of PFS and OS and log-rank tests were applied to evaluate differences in survival between groups. The fourth quartile was compared with the first, second, and third quartiles and to the combination of the lower 3 quartiles. Cox regression was used for assessing HRs between the fourth and the combination of the lower 3 quartiles in unadjusted analysis and in the multivariable model described above.

## RESULTS

### Patient Selection and Characteristics

Of the 103 patients enrolled in the ILUMINET trial, 6 were excluded because of previous PRRT, 3 were excluded because of progression or death immediately after inclusion, 12 patients were excluded because of nonanalyzable SPECT images, and 1 patient withdrew their consent. In total, 81 patients were included. For the analysis of the accumulated AD over all cycles, 1 additional patient was excluded because of nonanalyzable images for later cycles, making the number of patients included 80. The selection process is illustrated in Figure 1.

**FIGURE 1.**
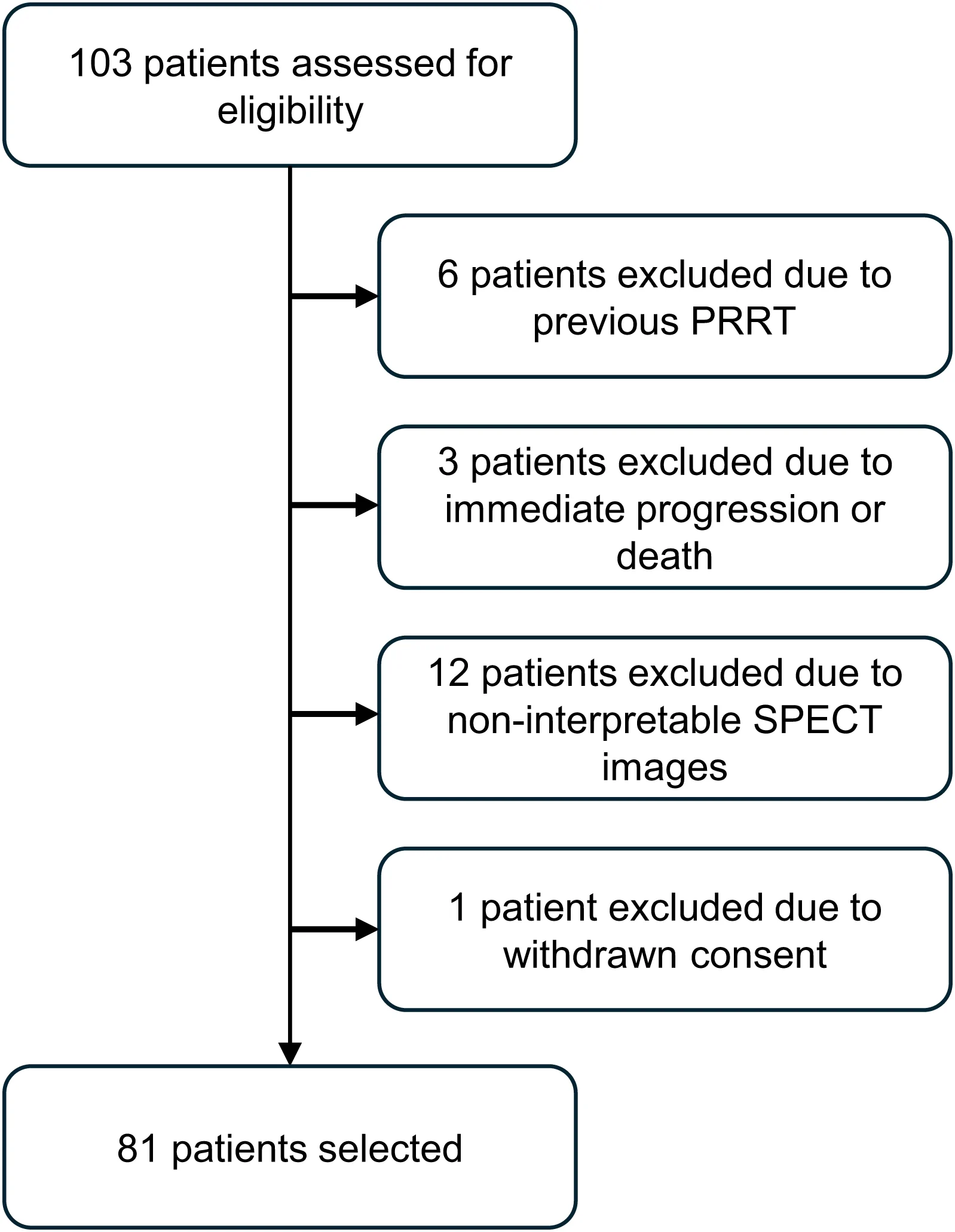
Schematic overview of patient selection process.

Baseline characteristics of the included patients are presented in Table 1. Most patients had an ECOG performance status of 0 or 1, with few classified as ECOG 2. A considerable proportion (41%) had only been treated with SSAs before PRRT. The most common number of non-SSA treatment lines received was 1 (27%), with progressively fewer patients having received multiple treatment lines. Small intestine was the most frequent tumor origin, followed by pancreas, while other sites were uncommon. Tumor grade and sex were evenly distributed between grades 1 and 2 and between sexes. Most patients received between 4 and 6 treatment cycles, with a few continuing beyond 7 cycles.

**TABLE 1.** Baseline Patient Characteristics for Total Cohort and Stratified by AD Group for Cycle 1 AD and Cumulative AD

		Cycle 1 AD group*		Cumulative AD group^†^	
Factor	Total cohort^‡^	Low AD	High AD	Low AD	High AD
Performance status					
ECOG 0	(56, 55)	24	32	24	31
ECOG 1	23	15	8	15	8
ECOG 2	2	1	1	1	1
Treatment lines					
0	33	17	16	17	16
≥1	(48, 47)	23	25	23	24
1	22	16	6	14	8
2	11	5	6	4	7
3	(9, 8)	2	7	3	5
4	3	0	3	1	2
5	3	0	3	1	2
Tumor origin					
Small intestine	53	30	23	29	24
Pancreas	(14, 13)	2	12	4	9
Lung	6	4	2	4	2
Colorectum	4	1	3	1	3
Other^§^	4	3	1	2	2
Tumor grade					
1	42	19	23	18	24
2	(39, 38)	21	18	22	16
Sex					
Male	(44, 43)	20	24	17	26
Female	37	20	17	23	14
Number of cycles					
2	6	2	4	5	1
3	12	10	2	11	1
4	14	8	6	8	6
5	20	8	12	8	12
6	19	8	11	5	14
7	4	1	3	1	3
8	(5, 4)	2	3	1	3
9	1	1	0	1	0
Median age (y)	66.61	68.63	64.71	66.65	65.72

* *n* = 81.

† *n* = 80.

‡ Total number of patients per factor; where numbers differ between cycle 1 AD and cumulative AD groups, respective group totals are indicated in parentheses.

§ Includes ventricle, duodenum, and unknown origins.

Previous treatment lines refer to anticancer treatments other than SSA.

### AD Distribution

In total, dosimetry was performed for 2 295 tumor VOIs, across all patients and all cycles. Overall, the median AD for cycle 1 was 19 Gy, whereas the median cumulative AD over all cycles was 74 Gy. Categorization based on these values yielded distributions between low- and high-AD groups that were broadly balanced across most of the baseline factors. Small intestinal NETs, however, were more common in the low-AD groups, whereas pancreatic NETs were more frequent in the high-AD groups (Table 1). Likewise, there was a tendency that patients in the low-AD group presented with performance status ECOG 1, whereas patients with ECOG 0 were slightly more frequent in the high-AD group. Tumor grade, prior treatment lines, and age showed no major imbalances between groups.

Table 2 shows the median ADs for cycle 1 and cumulatively across all cycles for the baseline factor subgroups. Notably, patients with pancreatic tumors consistently received higher ADs than those with small intestinal tumors. On a cohort level, there was a trend toward higher cumulative AD with an increasing number of cycles, although the dispersion around the median was considerable, reflecting the variation between patients (Fig. 2).

**TABLE 2.** Distribution of Cycle 1 AD and Cumulative AD Over Clinical Factors

Factor	Cycle 1 AD (Gy)	Cumulative AD (Gy)
Performance status		
ECOG 0	22 (4–63)	91 (12–265)
ECOG 1	16 (5–31)	51 (17–202)
ECOG 2	29 (11–47)	57 (30–83)
Tumor origin		
Small intestine	17 (4–53)	64 (12–265)
Pancreas	28 (5–63)	97 (17–194)
Lung	15 (6–24)	66 (35–130)
Colorectum	24 (14–35)	119 (49–174)
Other*	16 (11–28)	82 (50–142)
Tumor grade		
Grade 1	21 (6–63)	92 (21–265)
Grade 2	17 (4–55)	69 (12–209)
Sex		
Male	20 (6–47)	94 (23–265)
Female	17 (4–63)	57 (12–194)
Number of cycles		
2	20 (9–47)	36 (17–83)
3	12 (4–21)	38 (12–74)
4	15 (7–55)	59 (27–165)
5	23 (5–53)	88 (24–209)
6	23 (9–37)	115 (51–155)
7	27 (8–63)	192 (37–202)
8	19 (8–31)	138 (51–265)
9	6 (6–6)	63 (63–63)
Treatment lines		
0	18 (5–63)	73 (21–265)
1	14 (6–55)	54 (17–209)
2	26 (4–53)	91 (12–202)
3	24 (10–33)	96 (26–129)
4	23 (21–28)	78 (37–143)
5	26 (24–43)	90 (46–123)

* Includes ventricle, duodenum, and unknown origins.

Data are presented as median and range. Previous treatment lines refer to anticancer treatments other than SSA.

**FIGURE 2.**
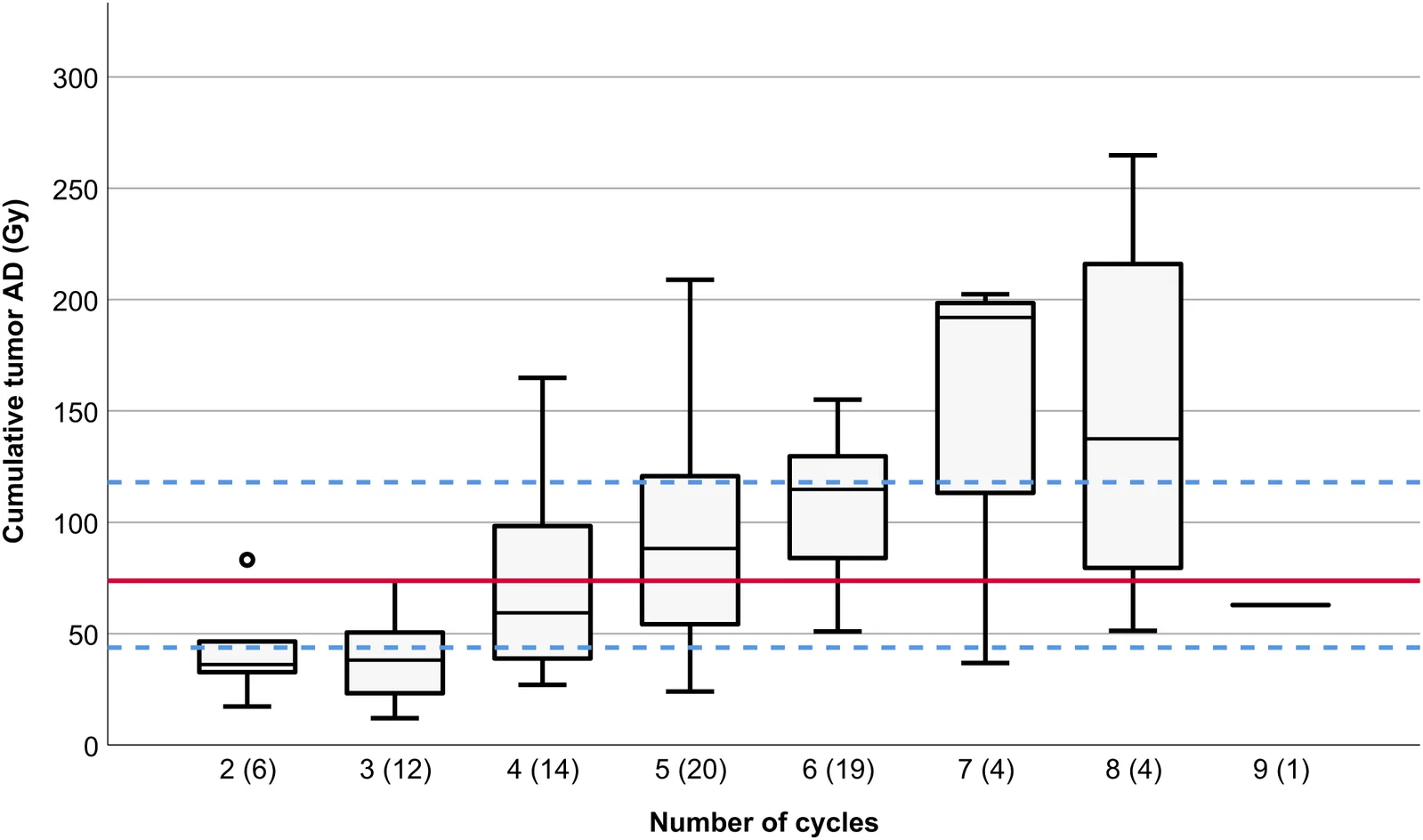
Distribution of cumulative tumor AD across treatment cycles, stratified by number of cycles received (2–9). Number in parentheses indicates patients per category. Solid red lines indicate total-cohort median AD (74 Gy), and dashed blue lines mark first (46 Gy) and third (118 Gy) quartile ADs. Boxes extend across interquartile range, whiskers extend to 1.5× interquartile range, and outlier is shown as individual marker.

### Relationship Between AD and Survival

Kaplan–Meier curves and log-rank tests showed significant survival benefits in terms of both PFS and OS for the patients in the high-AD groups for both the AD for cycle 1 and cumulative over all cycles (Fig. 3). This was confirmed in a univariable Cox regression in which patients in the high-AD groups had significantly lower HRs for PFS and OS than did the patients in the low-AD groups (Table 3). In the multivariable models for cycle 1 and cumulative ADs, the high-AD groups had significantly lower HRs for PFS and OS than did the patients in the low-AD groups. For cumulative ADs, the HR for PFS was 0.37 (95% CI, 0.21–0.66), whereas the HR for OS was 0.48 (95% CI, 0.25–0.93). Furthermore, not having received previous treatments other than SSA and having an ECOG performance status of 0 compared with a score of 1 or greater were independent markers of improved survival, whereas tumor grade and age were not (Fig. 4). The sensitivity analysis in which tumor grade was replaced by tumor origin rendered similar results (Supplement 3).

**FIGURE 3.**
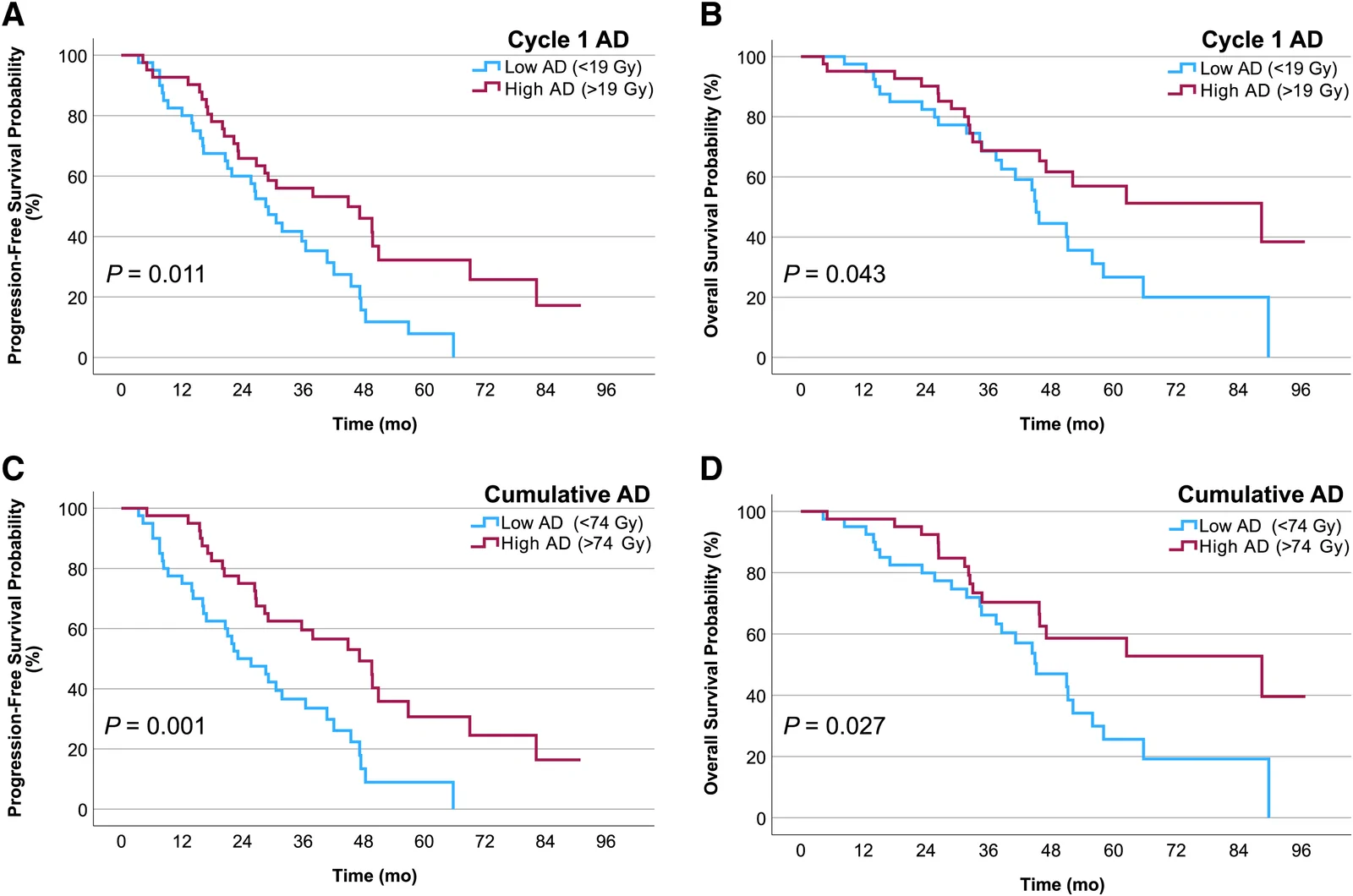
Kaplan–Meier curves of survival probability stratified at tumor AD higher or lower than total-cohort medians. (A) PFS for cycle 1 AD, (B) OS for cycle 1 AD, (C) PFS for cumulative AD, and (D) OS for cumulative AD. The total-cohort median for cycle 1 AD was 19 Gy, whereas for cumulative AD it was 74 Gy. *P* values are indicated.

**TABLE 3.** HRs Resulting from Univariable and Multivariable Cox Regression Analyses of PFS and OS, Both Stratified at Cohort Median Cycle 1 AD of 19 Gy and Cumulative AD of 74 Gy

	Cycle 1 AD		Cumulative AD	
	PFS HR	OS HR	PFS HR	OS HR
Univariable				
High tumor AD vs. low tumor AD	0.51 (0.30–0.86)*	0.53 (0.29–0.99)*	0.42 (0.24–0.73)**	0.50 (0.27–0.93)*
Multivariable				
High tumor AD vs. low tumor AD	0.34 (0.19–0.63)**	0.41 (0.21–0.81)*	0.37 (0.21–0.66)**	0.48 (0.25–0.93)*
Tumor grade 1 vs. grade 2	0.97 (0.56–1.70)	1.08 (0.56–2.08)	1.04 (0.61–1.80)	1.18 (0.62–2.27)
ECOG (0 vs. ≥1)	0.48 (0.27–0.85)*	0.47 (0.25–0.89)*	0.53 (0.30–0.94)*	0.49 (0.26–0.94)*
Previous treatment (no vs. yes)	0.38 (0.21–0.69)**	0.32 (0.16–0.67)**	0.40 (0.22–0.73)**	0.33 (0.16–0.69)**
Age (y)	0.95 (0.92–0.98)***	0.97 (0.94–1.00)	0.96 (0.93–0.98)**	0.98 (0.95–1.01)

HRs are presented with 95% CIs in parentheses.

Previous treatment refers to anticancer treatments other than SSA. *P < 0.05, **P < 0.005, and ***P < 0.0005.

**FIGURE 4.**
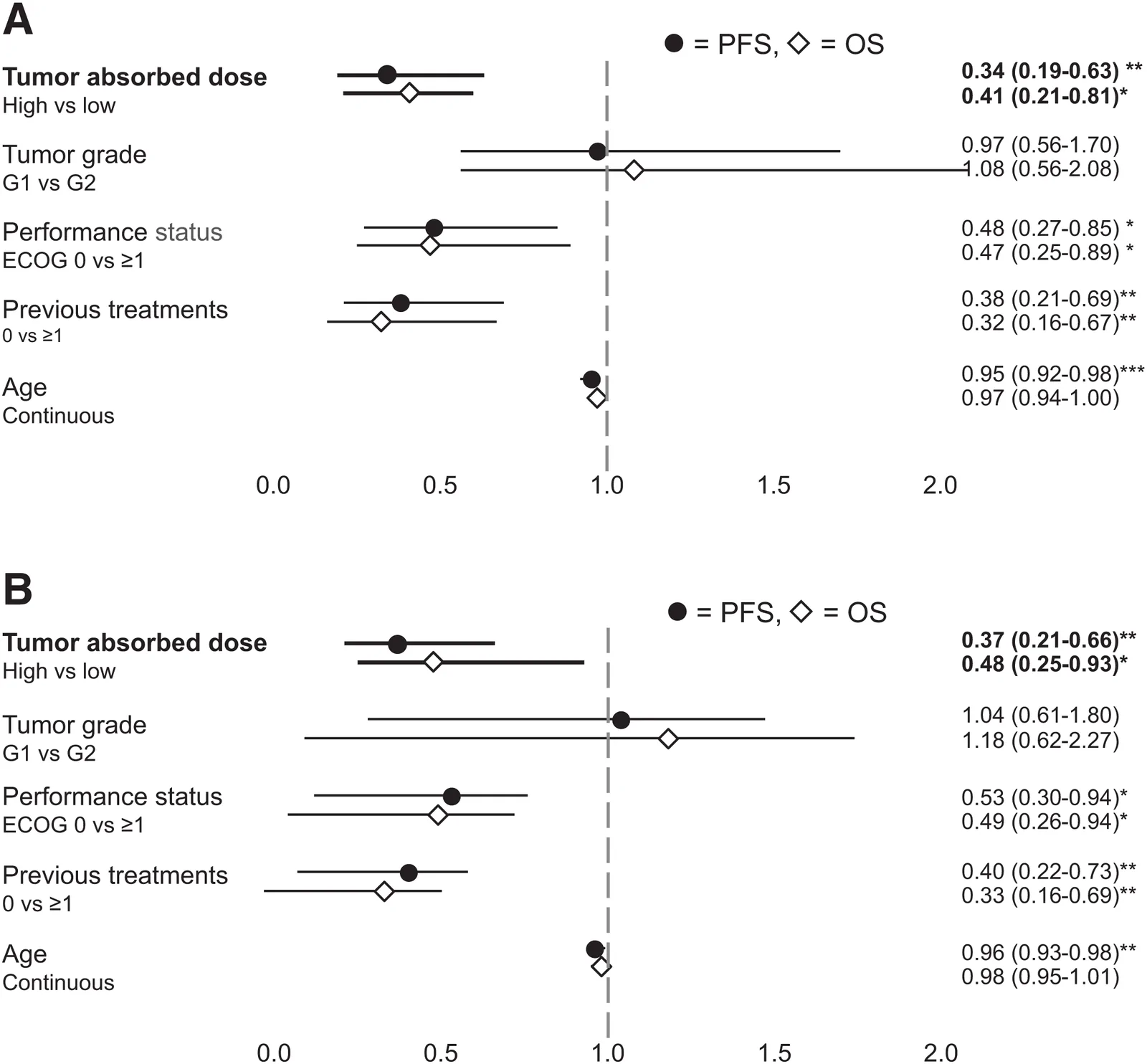
Forest plot displaying HRs (● = PFS, ◊ = OS) and 95% CI (whiskers) for variables in multivariable Cox regression. Analyses for tumor AD for cycle 1 and cumulatively across all cycles are shown in panels A and B, respectively. G1 = grade 1; G2 = grade 2.

Median PFS and OS were generally higher in the high AD groups, as displayed in Table 4. With regard to tumor origin, the number of patients with small intestinal NETs was sufficiently high to enable subgroup analyses, whereas the patients with pancreatic NETs were too few to allow for separate analyses. Kaplan–Meier analyses and log-rank tests demonstrated significant separations between the high- and low-AD groups (dichotomized at the median AD of 63 Gy for small intestinal NETs) for both PFS and OS using the cumulative AD and for PFS using the cycle 1 AD. Moreover, a high cumulative tumor AD was found to be an independent predictor of improved PFS and OS in multivariable analysis (Supplement 6).

**TABLE 4.** Estimated Median PFS and OS for Low-AD and High-AD Groups, Dichotomized at Cohort Median Cycle 1 AD and Median Cumulative AD

AD group	Median PFS (mo)	Median OS (mo)
Cycle 1 AD high (≥19 Gy)	47.1	88.4
Cycle 1 AD low (<19 Gy)	28.6	45.1
Cumulative AD high (≥74 Gy)	47.1	88.4
Cumulative AD low (<74 Gy)	23.1	45.1

### Identification of a Clinically Relevant Threshold for AD

Table 5 shows the AD quartiles, estimated median PFS, median OS, and the occurrence of adverse events. For the AD at cycle 1, Kaplan–Meier curves did not show any significant separations of the survival curves categorized into AD quartiles, although a slight trend toward improved survival with higher AD was observed (not shown). With respect to the cumulative AD, Kaplan–Meier analyses showed significantly improved PFS and OS for the fourth quartile (>118 Gy), compared with the first, second, and third quartiles and the combination of the first, second, and third quartiles, respectively (Fig. 5). There was no difference in frequency of high-grade toxicity between the quartiles.

**TABLE 5.** Estimated Median PFS and OS for Cohort Quartiles of Cumulative Tumor AD*

Quartile	*n*	AD (Gy)	Median PFS (mo)	Median OS (mo)	No. grade ≥3 AE
1	20	≤46	14.2	21.8	5
2	20	47–74	30.6	40.7	4
3	20	75–118	26.7	37.8	4
1–3	60	≤118	26.4	45.1	13
4	20	>118	50.9	Not reached	4

* Estimates for combined lower 3 quartiles (Q1–Q3) are also shown, as well as observed number of grade ≥3 adverse event (AE). Median OS was not reached in quartile 4. Patients were followed for median of 47 mo (interquartile range, 33–63 mo), with maximum follow-up of 97 mo. At 60 and 96 mo, 6 and 1 patients, respectively, remained under observation.

**FIGURE 5.**
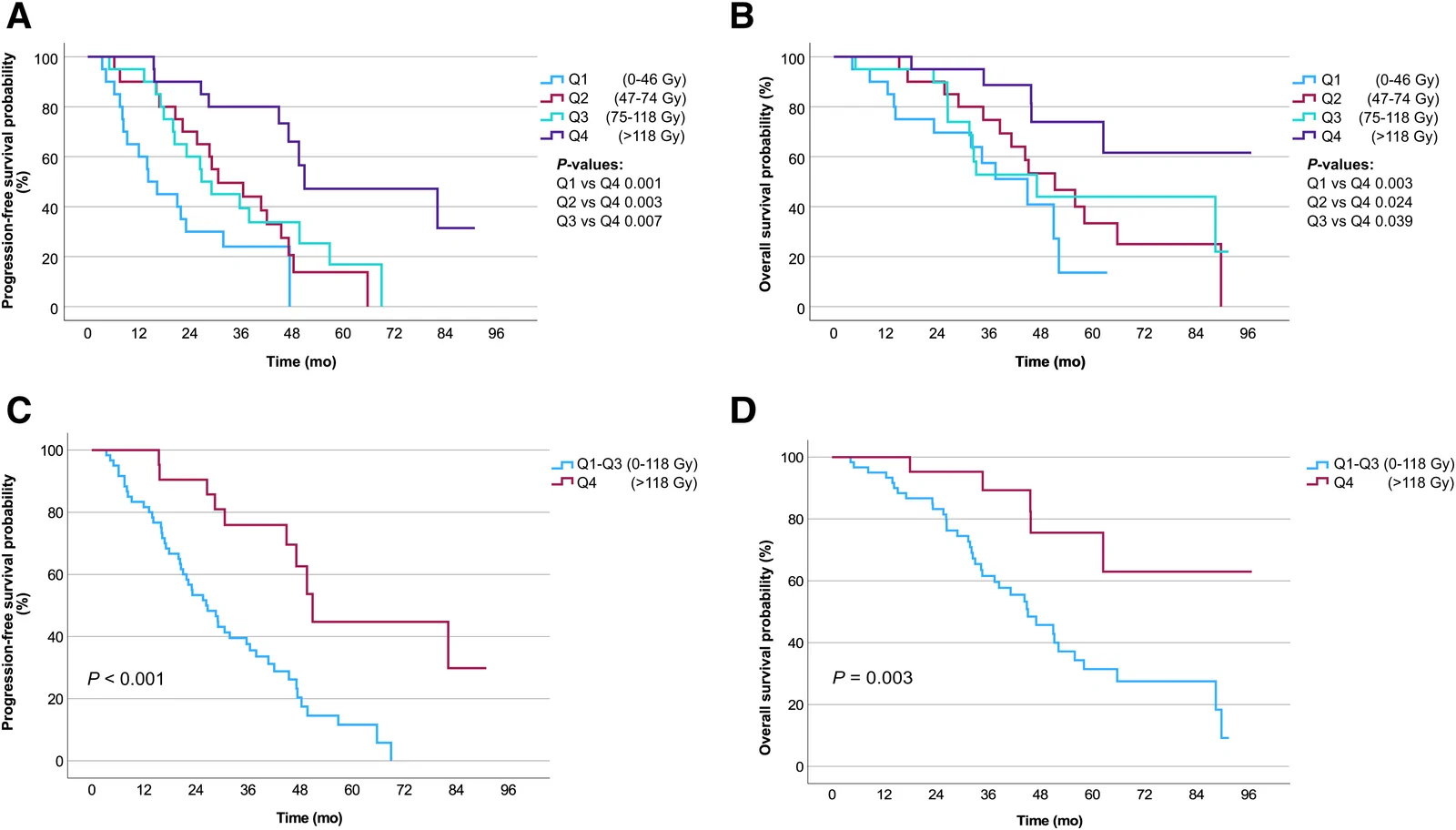
Kaplan–Meier curves of survival probability stratified at cohort quartiles (Q1–Q4) of cumulative tumor AD. (A) PFS with respect to cumulative AD, with <47 Gy for Q1 (blue), 47–74 Gy for Q2 (red), 75–118 Gy for Q3 (green), and >118 Gy for Q4 (purple). (B) OS for same quartiles. (C) PFS and (D) OS with respect to cumulative AD for highest quartile (Q4, >118 Gy, red) versus combined quartiles Q1–Q3 (≤118 Gy, blue). Numbers at risk are included for (C) and (D) and omitted for (A) and (B) for reasons of space. *P* values are indicated.

When a variable dichotomized on patients in the fourth quartile versus the remaining cohort in the multivariable model was included, cumulative mean AD was the strongest predictor of PFS and OS. Patients in the highest quartile had significantly lower HRs for both PFS (HR, 0.27; 95% CI, 0.13–0.59) and OS (HR, 0.28; 95% CI, 0.10–0.73), independent of prior treatment, ECOG performance status, tumor grade, and age (Table 6).

**TABLE 6.** Multivariable Cox Regression Analysis Comparing Patients in Highest Quartile of Cumulative AD (>118 Gy) to Rest of Cohort

Factor	PFS HR	OS HR
High tumor AD vs. low tumor AD	0.27 (0.13–0.59)**	0.28 (0.11–0.73)*
Tumor grade 1 vs. tumor grade 2	1.03 (0.60–1.78)	1.19 (0.63–2.27)
ECOG 0 or ≥1	0.52 (0.29–0.91)*	0.46 (0.24–0.88)*
Previous treatment (no vs. yes)	0.48 (0.27–0.84)*	0.34 (0.17–0.70)**
Age (y)	0.97 (0.95–1.00)*	0.98 (0.96–1.01)

HRs are presented with 95% CIs in parentheses, adjusted for tumor grade, ECOG performance status, previous treatment, and age. Previous treatment refers to anticancer treatments other than SSA. *P < 0.05, **P < 0.005.

## DISCUSSION

In this post hoc analysis of data collected within a prospective phase 2 study, we show that mean AD to the entire tumor burden is an independent positive predictor of survival in patients with grades 1 and 2 NETs treated with [^177^Lu]Lu-DOTATATE. Although previous studies have demonstrated dose–survival relationships, none have, to the best of our knowledge, confirmed this association through adjusted survival analyses and estimation of the AD to the whole tumor burden.

Survival improves progressively with increasing tumor AD, as demonstrated when separated into low- and high-AD groups (Fig. 3) and by quartile-based analysis (Fig. 5). Identifying dose–response relationships is a key criterion for establishing causality (21) and thus supports the interpretation that tumor AD is not merely associated with survival but suggests that tumor AD may be a key determinant of therapeutic efficacy in PRRT. Specifically, our findings suggest that a tumor AD of at least 118 Gy is associated with improved survival outcomes without any observable increase in toxicity.

This threshold of 118 Gy is comparable to the result from previous studies. Hebert et al. (10) performed dosimetry for up to 5 selected lesions in the liver, lymph nodes, mesenteric mass, pancreas, and peritoneum and calculated each patient’s mean tumor AD. They showed a significantly improved PFS at mean tumor ADs above 91.36 Gy, with a trend toward improved OS. Mileva et al. (12) observed prolonged PFS when the tumor receiving the lowest AD exceeded 35 Gy, which, when extrapolated to 4 treatment cycles, could result in cumulative tumor AD in a range corresponding to that of other studies, although cumulative tumor AD estimates remain uncertain because of potential reduction across cycles (18). Ebbers et al. (22) proposed a threshold of 101 Gy (measured as the mean tumor AD over up to 5 selected liver lesions) for disease stabilization after selective internal radiotherapy with ^90^Y-loaded microspheres.

In our study, of the patients receiving 118 Gy or higher, 14 of 20 patients needed more than the standard 4 cycles to reach 118 Gy (Supplement 5). Regarding cumulative renal biologically effective dose, none of these patients had a cumulative renal biologically effective dose exceeding 28 Gy (Supplement 5).

On the other hand, other proposed thresholds deviate more substantially. Hebert et al. (10) also dichotomized patients on the basis of the AD of the tumor with the lowest AD. Patients whose lowest-dose tumor AD exceeded 52.52 Gy had significantly longer PFS and OS than those below this threshold. Maccauro et al. (11) focused on the mass-weighted mean AD across all lesions after the first treatment cycle. They found that patients with a mean AD greater than 10.6 Gy had significantly longer PFS than patients with a mean AD below 10.6 Gy.

As outlined above, the available studies use diverse methodologies for assessing tumor AD during PRRT. This heterogeneity poses a significant challenge to cross-study comparisons and limits the generalizability of dose–response findings. As evidence linking tumor AD to survival continues to grow, the next critical step for clinical translation is to define a standardized measurand for tumor dosimetry. This includes determining which lesions should be selected and whether the tumor AD should be based on the minimum, arithmetic mean, mass-weighted mean, or another metric. Establishing such protocols would also enable broader application of dosimetric methods.

Furthermore, there is a need for consensus protocols for tumor identification and its relevant imaging support from PET, SPECT, contrast-enhanced CT, or MRI. In this study, we chose to base tumor identification mainly on SPECT images acquired during ^177^Lu-labeled PRRT, under the assumption that viable tumors also exhibit somatostatin expression and [^177^Lu]Lu-DOTATATE uptake, however, with the risk of falsely omitting tumors with limited uptake. Contrast-enhanced CT, MRI, and [^68^Ga]Ga-DOTA-SSTR PET images were mainly used for confirmation.

A limitation of the present study is the use of fixed effective half-lives for grade 1 and grade 2 NETs. Given that several studies have demonstrated relatively stable effective half-lives (18,23,24), this was adopted as a pragmatic solution to enable tumor AD estimation in the absence of multiple intracycle SPECT acquisitions. On the other hand, tumor ADs were evaluated for each treatment cycle, enabling analyses of both cycle 1 tumor ADs and the cumulative ADs across all treatments. Another limitation of this study is its post hoc design, based on prospectively collected data from a phase 2 trial. Although the structured data collection improves the quality of the dataset, analyzing it retrospectively may still introduce bias. Nevertheless, our findings support the underlying premise of a clinically meaningful association between tumor AD and patient outcomes and further reinforce recent calls for prospective PRRT trials incorporating individualized treatment planning through tumor dosimetry (25).

## CONCLUSION

This post hoc analysis of prospectively collected data from a phase 2 trial demonstrates that mean tumor AD—both in cycle 1 and cumulatively throughout the course of treatment—is an independent predictor of PFS and OS in patients with grade 1 or grade 2 NETs treated with [^177^Lu]Lu-DOTATATE. Both PFS and OS improved progressively with increasing tumor AD, and a cumulative mean AD of approximately 118 Gy was associated with significantly better outcomes without increased toxicity. These findings underscore the clinical relevance of tumor dosimetry and support its integration into future prospective PRRT trials aimed at individualized treatment planning.

## DISCLOSURE

Katarina Sjögreen Gleisner wishes to acknowledge financial support from the Mrs. Berta Kamprad’s Cancer Foundation (GN FBKS-2024-7) and the Swedish Cancer Society (GN 24 3785 Pj 01 H). Johan Gustafsson acknowledges funding from the Mrs. Berta Kamprad’s Foundation (FBKS-2025-21-689). Carl Warfvinge has received advisory board fees from Ipsen and speaker honorarium from AstraZeneca. No other potential conflict of interest relevant to this article was reported.

## Notes

KEY POINTS **QUESTION:** Is an increased tumor AD associated with increased survival in patients with NETs treated with [^177^Lu]Lu-DOTATATE? **PERTINENT FINDINGS:** In a study of 81 patients treated with [^177^Lu]Lu-DOTATATE, a cumulative mean absorbed tumor dose over median (74 Gy) and a mean AD in cycle 1 over median (19 Gy) were independent predictors of prolonged PFS and OS. Patients receiving a cumulative AD above approximately 118 Gy had significantly better outcomes without increased toxicity. **IMPLICATIONS FOR PATIENT CARE:** These findings support the integration of tumor dosimetry into treatment planning for PRRT. Targeting a cumulative AD of at least 118 Gy may improve survival outcomes and enable more individualized therapy without compromising safety.
